# Resveratrol increases F508del-CFTR dependent salivary secretion in cystic fibrosis mice

**DOI:** 10.1242/bio.010967

**Published:** 2015-06-19

**Authors:** Barbara Dhooghe, Charlotte Bouckaert, Arnaud Capron, Pierre Wallemacq, Teresinha Leal, Sabrina Noel

**Affiliations:** 1 Louvain Centre for Toxicology and Applied Pharmacology, Institut de Recherche Expérimentale et Clinique, Université Catholique de Louvain, Brussels B1200, Belgium; 2 Clinical Chemistry Department, Cliniques Universitaires St. Luc, Université Catholique de Louvain, Brussels B1200, Belgium

**Keywords:** Cystic fibrosis, Mouse model, CFTR, Salivary secretion, Pharmacology

## Abstract

Cystic fibrosis (CF) is a fatal genetic disease associated with widespread exocrine gland dysfunction. Studies have suggested activating effects of resveratrol, a naturally-occurring polyphenol compound with antioxidant and anti-inflammatory properties, on CF transmembrane conductance regulator (CFTR) protein function. We assayed, in F508del-CFTR homozygous (CF) and in wild-type mice, the effect of resveratrol on salivary secretion in basal conditions, in response to inhibition by atropine (basal β-adrenergic-dependent component) and to stimulation by isoprenaline (CFTR-dependent component). Both components of the salivary secretion were smaller in CF mice than in controls. Two hours after intraperitoneal administration of resveratrol (50 mg/kg) dissolved in DMSO, the compound was detected in salivary glands. As in both CF and in wild-type mice, DMSO alone increased the response to isoprenaline in males but not in females, the effect of resveratrol was only measured in females. In wild-type mice, isoprenaline increased secretion by more than half. In CF mice, resveratrol rescued the response to isoprenaline, eliciting a 2.5-fold increase of β-adrenergic-stimulated secretion. We conclude that the salivary secretion assay is suitable to test DMSO-soluble CFTR modulators in female mice. We show that resveratrol applied *in vivo* to mice reaches salivary glands and increases β-adrenergic secretion. Immunolabelling of CFTR in human bronchial epithelial cells suggests that the effect is associated with increased CFTR protein expression. Our data support the view that resveratrol is beneficial for treating CF. The salivary secretion assay has a potential application to test efficacy of novel CF therapies.

## INTRODUCTION

Cystic Fibrosis (CF), a complex disease associated with widespread exocrine gland dysfunction, is caused by loss of function of the CF transmembrane conductance regulator (CFTR) chloride channel. Almost 2000 mutations have been described, but ∼70% of patients carry at least one F508del-CFTR mutation. The deletion of the phenylalanine at position 508 of CFTR impairs the folding of the protein which is retained in the endoplasmic reticulum and undergoes early degradation by the proteasome. The loss of CFTR contribution to ion homeostasis drives an impaired mucociliary clearance and promotes cycles of chronic inflammation and bacterial infection. Therefore, CF patients would strongly benefit from pharmacological compounds combining the ability to correct both the deficient ion transport and the deregulated inflammatory responses.

Resveratrol, a natural polyphenolic antioxidant abundant in grapes and pomegranates with claimed cardioprotecting (Wu and Hsieh, 2011) and cancer preventing (Bhat and Pezzuto, 2002) properties, has drawn growing scientific attention. Anti-inflammatory properties, that would be beneficial in CF, have also been described (Alexander et al., 2011; Donnelly et al., 2004). An *in vivo* study (Alexander et al., 2011) showed acute stimulation of CFTR-mediated chloride transport across mouse nasal epithelium by resveratrol. It has been suggested that resveratrol potentiates CFTR channel activity through a cAMP-independent mechanism (Yang et al., 2013); however its molecular target is not clear. The fact that resveratrol may combine potentially beneficial effects on CFTR-mediated chloride transport and on airway inflammation justifies looking further into its effects on secretory epithelia in CF.

It was suggested more than 50 years ago that salivary glands (Barbero and Sibinga, 1962; Shackleford and Bentley, 1964) are altered in CF patients. Morphological changes consisting in enlarged ducts, dilated acini and thinner epithelial walls consistent with abnormal mucus plugs were identified in salivary glands from CF patients. It is well established that saliva is produced in response to both sympathetic and parasympathetic agonists that drive the coordinated activity of multiple water and ion transporters (Melvin, 1999; Melvin et al., 2005). An altered composition of saliva, including increased calcium and phosphorus contents, has been recognized in CF patients (Boat et al., 1974; Mandel et al., 1969).

In 2005, Best and Quinton developed a salivary secretion assay in mouse (Best and Quinton, 2005). They showed that salivary glands of mice knocked out for the CFTR protein (*cftr*^−/−^) failed to respond to the β-adrenergic agonist isoprenaline, while the cholinergic induced secretion remained intact. Another *in vivo* study showed that CFTR potentiators (MPB-07, genistein) increased salivary secretion in wild-type but not in *cftr*^−/−^ mice, highlighting the potentiality of this method to test the preclinical efficacy of CFTR modulators (Noel et al., 2008). The fact that only water and ethanol soluble compounds were used in this study (Noel et al., 2008) is quite important: in drug discovery programs, compounds are primarily synthesized to optimize their pharmacological activity, often resulting in highly lipophilic compounds with poor water solubility. Most of them require hydrophobic solvents, such as DMSO which can act as a chemical chaperone for CFTR (Planas et al., 2012). However, possible unwanted effects of DMSO on the salivary secretion assay have not been evaluated.

This study was designed to test the hypothesis that resveratrol stimulates *in vivo* salivary secretory responses in the presence of the F508del-CFTR protein. We showed in controlled experiments that detectable levels of resveratrol are achieved in salivary glands two hours after a single intraperitoneal injection of the compound and that it partially restores the refractory β-adrenergic response of salivary glands in CF female mice. The effect seemed to be associated with increased CFTR expression. The data show that the salivary secretion assay is suitable for testing the efficacy of DMSO-soluble compounds for CF therapies.

## RESULTS

### Saliva secretion in untreated mice

No adverse events occurred in any experimental groups. As lower rates of salivary secretion in female mice than in male have previously been reported in C57BL/6 (Best and Quinton, 2005) and in other backgrounds (Droebner and Sandner, 2013), we first analyzed the influence of genotype and sex on the different components of the salivary secretion in FVB/129 mice. Thus, between-group comparisons were performed with data obtained from the basal salivary secretion, the atropine-insensitive component and the isoprenaline-induced component.

**Fig. 1.**
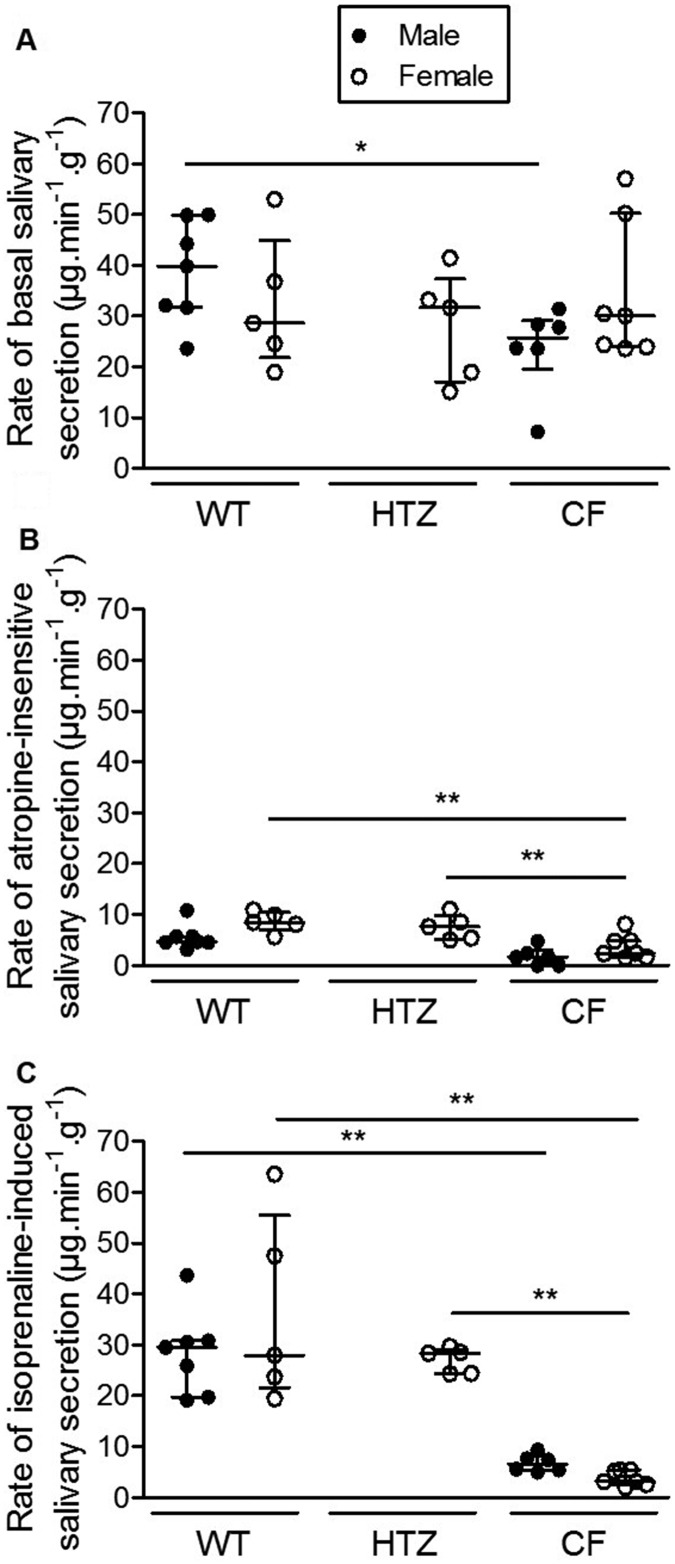
**Salivary secretion rate in F508del-CFTR homozygous mice (CF; male: *n*=6; female: *n*=7), heterozygous mice (HTZ; female: *n*=5) and wild-type mice (WT, male: *n*=7; female: *n*=5).** (A) Basal secretion tested in the absence of any pharmacological agent. (B) Atropine-insensitive secretion. (C) β-adrenergic stimulated salivary secretion assessed after injection of isoprenaline. Dark circles: males. Open circles: females. Data presented as median (larger horizontal line of datasets) and 25-75% interquartile ranges (lower and upper horizontal lines of datasets). Results normalized by animal body weight.*P<0.05; **P<0.01.

The average rate of basal salivary secretion tested in the absence of any agonist or inhibitor did not differ among the different genotypes, and similar rates were found in females and in males (Fig. 1A). In CF males, the average rate of basal secretion was significantly reduced to two thirds of the value found in wild-type males. The possible influence of body weight, significantly larger in male mice than in female (Table 1), was normalized by correcting rates of salivary secretion for animal body weight.

**Table 1.**
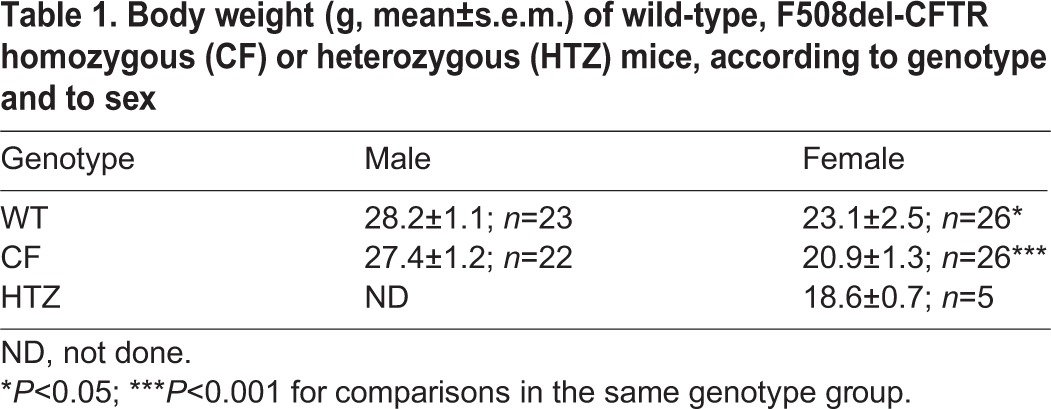
**Body weight (g, mean±s.e.m.) of wild-type, F508del-CFTR homozygous (CF) or heterozygous (HTZ) mice, according to genotype and to sex**

To isolate the β-adrenergic-sensitive fraction of the basal salivary secretion, the predominant cholinergic component of the salivary secretion was initially blocked with atropine (Fig. 1B). The average atropine-insensitive component of saliva secretion was significantly reduced by at least 50% in the CF group compared to the wild-type and the heterozygous groups. In heterozygous female mice, values significantly different from CF (*P*<0.05) but not from wild-type mice were observed (Fig. 1B). Integrity of the β-adrenergic-induced component was then tested by assessing the magnitude of the response to the β-agonist isoprenaline (Fig. 1C). In wild-type mice, a three- to four-fold increase in saliva secretion rate was observed after isoprenaline stimulation. This response was completely abolished in CF females as the median (25–75% interquartile range) obtained after β-adrenergic stimulation [3.2 (2.6–5.4) µg.min^−1^.g^−1^; Fig. 1C] did not significantly differ from the corresponding atropine-insensitive value [2.4 (1.7–4.9) µg.min^−1^.g^−1^; Fig. 1B]. However, a significant sensitivity to isoprenaline was detected in CF males: the average rate of isoprenaline-induced response in CF males was increased 4-fold after stimulation (*P*<0.05, compare Fig. 1B and 1C). However, beta-adrenergic-stimulated saliva secretion rates in CF males represented a small fraction (∼20%) of the corresponding values observed in wild-type mice (Fig. 1C). These data confirm that salivary secretion is altered in CF mice: the predominant atropine-sensitive component is intact and apparently not influenced by sex, while the remaining atropine-insensitive fraction is reduced and refractory to β-agonist stimulation, particularly in females.

### Effect of DMSO

Prior to evaluating the effect of resveratrol on salivary secretion in CF mice, control experiments were performed to test the effect of DMSO alone. Basal salivary secretion, atropine-insensitive secretion and isoprenaline-induced secretion were evaluated two hours after intraperitoneal injection of DMSO:NaCl (1:1) in CF and in wild-type mice of both sexes. Median rates of basal salivary secretion (tested in the absence of any agonist or inhibitor) and of atropine-insensitive secretion (tested under cholinergic blockade with atropine) were not influenced by DMSO (data not shown). However, distinct sex-dependent effects of DMSO were observed on the median rate of isoprenaline-stimulated salivary secretion (Table 2). The vehicle did not modify isoprenaline-stimulated salivary secretion in females. However, in males, a significant response to isoprenaline was observed two hours after injection of the vehicle.

**Table 2.**
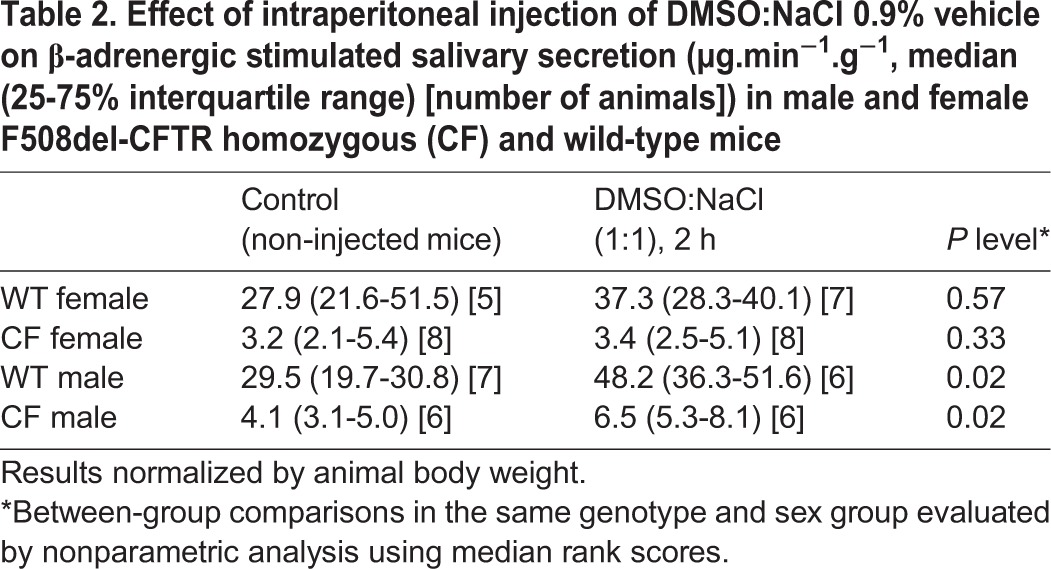
**Effect of intraperitoneal injection of DMSO:NaCl 0.9% vehicle on β-adrenergic stimulated salivary secretion (µg.min^−1^.g^−1^, median (25-75% interquartile range) [number of animals]) in male and female F508del-CFTR homozygous (CF) and wild-type mice**

### Response to resveratrol

In order to correspond to the delay of *in vivo* salivary tests (pharmacodynamics effects), pharmacokinetic studies were performed two hours after intraperitoneal administration of resveratrol. Plasma and salivary gland concentrations are given in Table 3. Concentration values at the limit of detection (0.5 ng/ml) were found in plasma samples from 3 out of 4 mice. In salivary glands, the median concentration was about 30 times larger than in plasma. Typical ion chromatograms of sample extracts obtained from a blank sample, an internal standard or from a sample spiked at the 1 ng/ml are shown in supplementary material Fig. S1. Chromatographs of a blood sample and of a salivary gland sample at 26.6 ng/ml and 27.4 ng/mg resveratrol respectively are also shown (supplementary material Fig. S1).

**Table 3.**
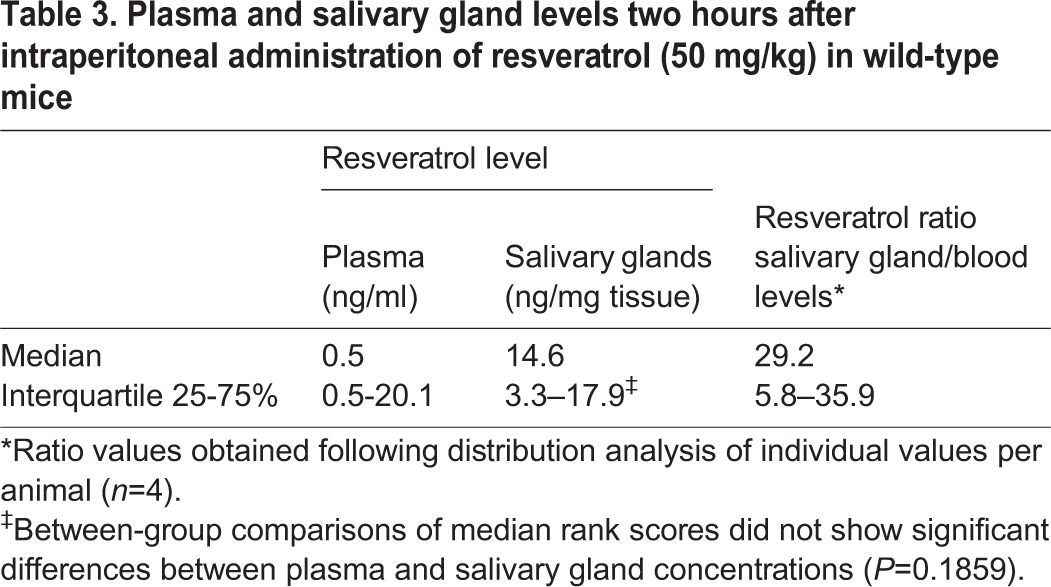
**Plasma and salivary gland levels two hours after intraperitoneal administration of resveratrol (50 mg/kg) in wild-type mice**

**Fig. 2.**
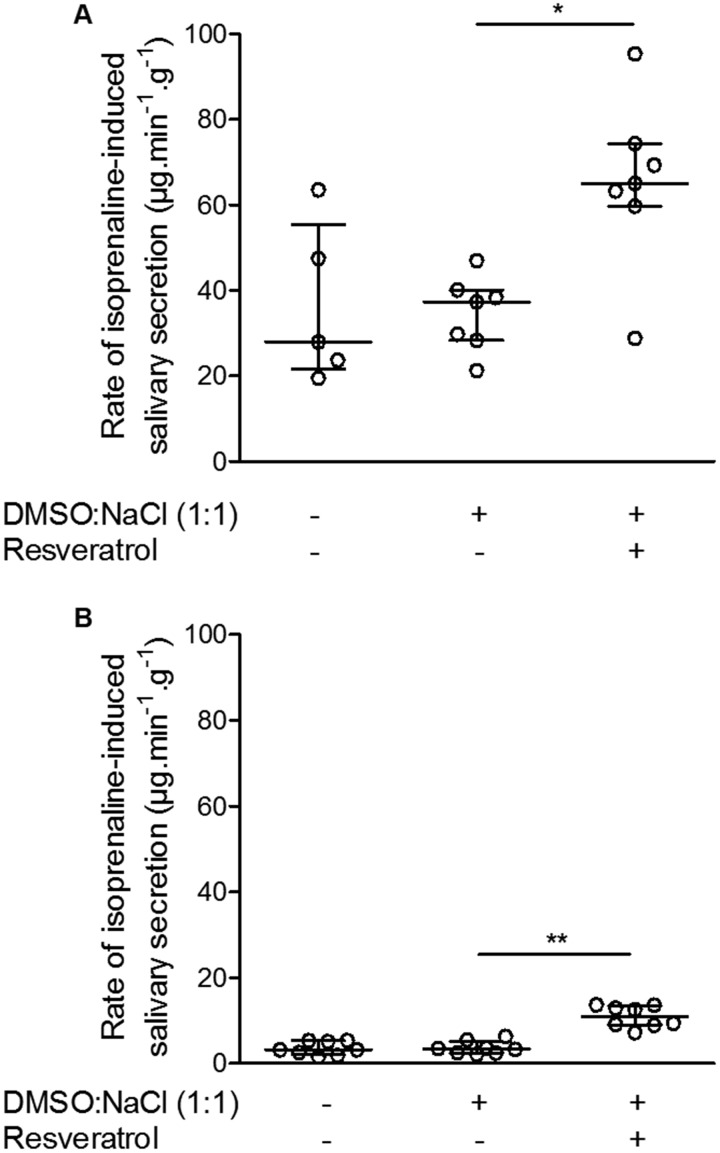
**Rate of β-adrenergic stimulated salivary secretion in female mice two hours after intraperitoneal injection of resveratrol versus DMSO:NaCl vehicle.** (A) Wild-type mice (DMSO:NaCl, *n*=7; resveratrol, *n*=7). (B) F508del-CFTR homozygous mice (DMSO-NaCl, *n*=8; resveratrol, *n*=8). For comparison, response in non-injected wild-type and CF control animals are also illustrated. Data presented as median (larger horizontal line of datasets) and 25-75% interquartile range (lower and upper horizontal lines of datasets). Results normalized by animal body weight.*P<0.05; **P<0.01.

In DMSO-controlled experiments, we tested the effect of resveratrol on salivary secretion in CF and wild-type mice. Because the response to isoprenaline was largely refractory and no effect of DMSO was observed in CF females, only female mice were used to evaluate the effect of resveratrol. In wild-type mice, the effect of resveratrol on the rate of isoprenaline-induced saliva secretion was increased by more than half compared to that measured in the DMSO controlled group (Fig. 2A). In CF mice, resveratrol partially restored the response to isoprenaline, eliciting a 2.5-fold increase of the rate of β-adrenergic-stimulated salivary secretion (Fig. 2B).

In no case did resveratrol modify basal secretion and atropine-insensitive secretion (data not shown).

### Influence of Resveratrol on CFTR mRNA and protein expression in human bronchial epithelial cells

**Fig. 3.**
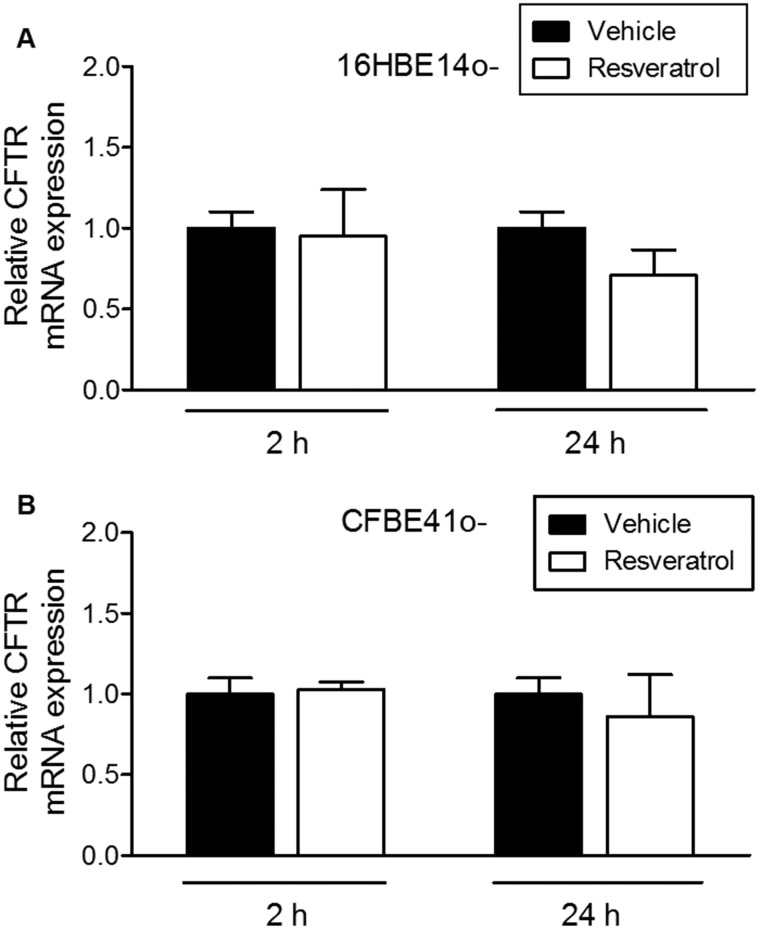
**Quantification of CFTR mRNA levels by RT-qPCR.** Relative expression of CFTR mRNA 2 h and 24 h after incubation of wild-type 16HBE14o− cells (A) and CFBE41o− (B) with resveratrol. Data are expressed as mean±s.e.m. No significant differences found.

**Fig. 4.**
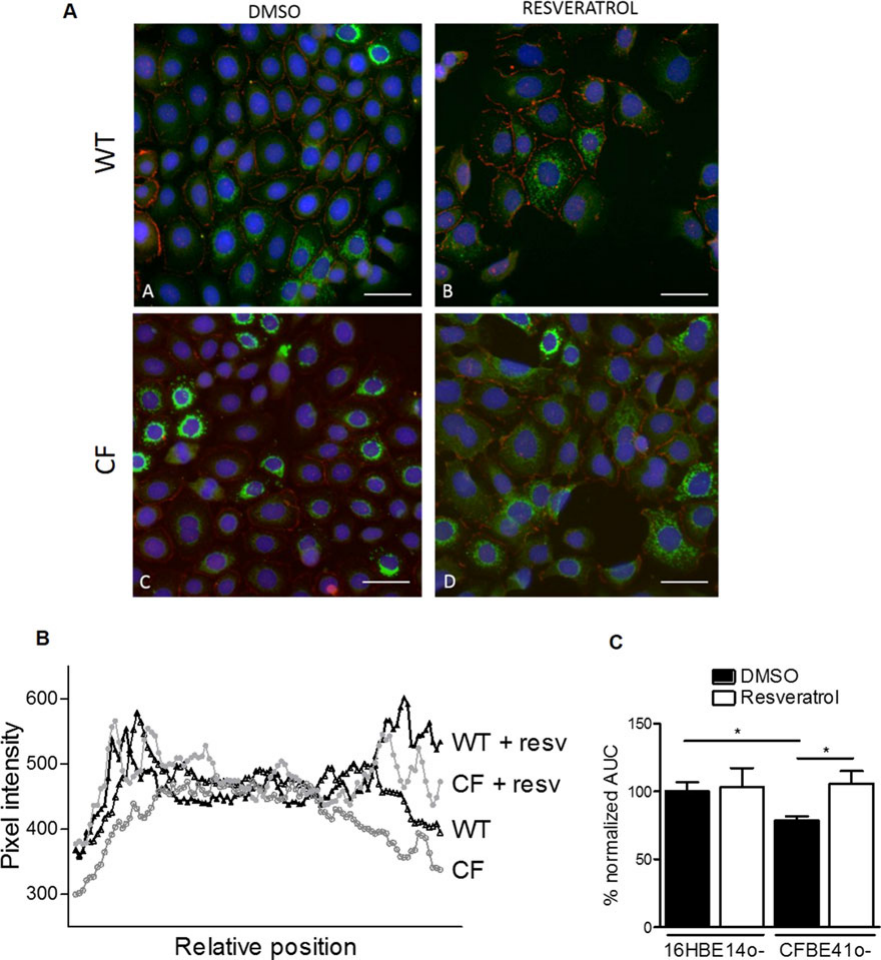
**Quantification of CFTR protein by immunofluorescence in human bronchial epithelial cells.** (A) Representative immunohistochemical labeling of CFTR (green) and zonula occludens (ZO-1, red) 24 h after incubation with DMSO (left) or 100 µM resveratrol (right) in wild-type 16HBE14o− (WT, top) or in CFBE41o− (CF, bottom) cultures. Nuclei (blue) stained by DAPI. Bar: 20 µm. (B) Relative position corresponding to intensity of the CFTR signal quantified by morphometric analysis of cross-sectional profiles in individual (*n*=8 cells per condition, randomly selected). (C) Average area under the fluorescence curves (±s.e.m.) obtained in B normalized to that measured in DMSO-treated wild-type cells and expressed as a measure of total CFTR protein expression (*P<0.05, Student *t*-test). Data from a representative experiment selected from a series of 3 experiments with similar results. Resv, Resveratrol.

To assess the potential mechanism involved in the activating effect of resveratrol on CFTR-dependent salivary secretion, we examined the cellular expression of CFTR mRNA and protein in human immortalized CF and wild-type bronchial cells after incubation with the compound. No significant changes in CFTR mRNA expression were found in 16HBE14o− (Fig. 3A) or CFBE41o− cells (Fig. 3B) after 2 h or 24 h exposure to resveratrol. This finding suggests that resveratrol does not act by altering mRNA. We then tested the effect of the compound on the protein expression by immunohistochemical analysis. In DMSO-treated wild-type and CF cells examined in confluent HBE cells cultures, the fluorescence labelling was limited to a perinuclear ring, hardly any signal being observed in more peripheral areas of the cytoplasm. By contrast, in resveratrol-treated wild-type confluent cultures, a perinuclear ring was less clearly visible, and the fluorescence signal tended to spread out, leading to a diffuse labelling throughout the whole cytoplasm (Fig. 4, compare panels A and B). Likewise, in CF cells treated with resveratrol, the same differences were observed in the localization of the perinuclear versus peripheral labelling, but in addition, the intensity of the whole fluorescence signal was increased (Fig. 4A, compare panels C and D). To improve data analysis, we performed morphometric studies of the images. The immunohistochemical results were quantified using the subcellular method described by Pasyk et al. (Pasyk et al., 2015). Briefly, cross-sectional scans of pixel intensities of individual cells were measured at their largest diameter and analyzed using AxioVision software (version 4.9.1). The mean CFTR-related intensity across each cell (*n*=8 cells per condition, randomly selected) was measured as representative of CFTR distribution. This allowed confirming that CFTR overall expression is significantly decreased in CF cells. Indeed, CF cells showed a decreased normalized area under the curve (see Fig. 4C). After incubation in the presence of resveratrol, CF cells showed increased CFTR-related global (peripheral and perinuclear) fluorescence intensity. It did not significantly affect the global intensity of the signal in wild-type cells. In addition, redistribution of the fluorescence intensity signal towards cell membranes was observed even in wild-type cells. Redistribution of CFTR with a larger intensity of the signal close to membrane areas colocalized with zonula occludens ZO-1, used as dual labelling. As conclusions, in CF cells, resveratrol increased both the intensity of the whole fluorescence signal and the modified the localization of the perinuclear versus peripheral CFTR labelling. These findings suggest that the increased rate of isoprenaline-induced salivary secretion in CF salivary glands after resveratrol treatment could be associated with an increased CFTR protein expression.

## DISCUSSION

Developing pharmacological therapies to rescue mutated CFTR processing and function has been the goal of an increasing number of drug discovery programs over the past decade (reviewed in Becq et al., 2011). *In vitro* screening methods have led to the discovery of strong hits. However, these methods do not predict future *in vivo* efficacy or potential adverse effects in human. Animal testing is needed, and the mouse is the only model currently available for routine drug testing in CF. Like every animal model of human disease, the CF mouse model has been criticized, mainly based on the lack of spontaneous lung disease, most prominent in CF patients. However, it does recapitulate several phenotypical features of the human disease. Indeed, F508del-CFTR mice present typical transepithelial ion transport abnormalities (van Doorninck et al., 1995) as we (Lubamba et al., 2009, 2008) and others (Droebner and Sandner, 2013; Saussereau et al., 2013) have demonstrated by measuring nasal potential difference. The failure of salivary glands to respond to β-adrenergic stimulation is an additional defect recapitulated in CF mice. Using normal and *cftr^−/−^* mice in a C57BL/6 background (CFTR^tm1Unc^), Best and Quinton have developed a rapid and straightforward method to quantify salivary secretion. Importantly, the method allows isolating the β-adrenergic component from the largely predominant cholinergic response (Best and Quinton, 2005), and thereby to test its integrity. The method was further used in wild-type mice to measure the effects of local injections of CFTR activators such as MPB-07 and genistein (Noel et al., 2008), highlighting its application to conduct preclinical *in vivo* studies on CFTR activity. Homozygous F508del-CFTR mice similar to those used in our work have already been studied to test the corrector effect of DMSO-soluble glafenine, an anthranilic acid derivative (Robert et al., 2010). Our current study provides additional control experiments; indeed we determined (1) the effects of the F508del-CFTR mutation relative to the FVB/129 wild-type mouse; (2) a possible influence of sex; and (3) the effect of the DMSO vehicle, all being crucial to validate this technique as a preclinical assay to study CFTR function in this CF mouse model.

In this study, we determined the salivary secretion rate in wild-type and F508del-CFTR homozygous and heterozygous mice. As originally reported for *cftr*^−/−^ mice (Best and Quinton, 2005), atropine drastically inhibited secretion in all CFTR genotypes. The atropine-insensitive secretion was lower in CF than in wild-type mice of both sexes although it did not reach significance in male. Isoprenaline increased secretion rate in wild-type animals, but not significantly in CF mice.

An influence of sex, probably dependent on the genetic background, on the β-adrenergic component of secretion has been previously reported (Best and Quinton, 2005; Droebner and Sandner, 2013). Here we found that basal salivary secretion tended to be lower in CF male mice compared to wild-type animals, but the difference was not observed in female groups. In contrast with Best and Quinton's findings on the C57BL/6 background, our results do not suggest a major influence of sex on the different components of salivary secretion in FVB/129 wild-type animals. However, we show here that CF female mice seem to be completely refractory to isoprenaline stimulation, whereas a significant response was evident in male mice, though the response was dramatically reduced relative to wild-type animals (compare Fig. 1B and 1C). Our data are in line with those obtained in other secretory glands, such as sweat glands, that may also be influenced by sex as recently shown in CF patients (Quinton et al., 2012).

Interestingly, we also found an influence of sex on the effect of DMSO, an epithelial differentiating agent that has been suspected to have a corrector effect on F508del-CFTR maturation defect (Bebök et al., 1998). Accordingly, it has been shown in a porcine kidney cell line, LLC-PK1, that insertion of functional F508del-CFTR to the plasma membrane was significantly increased four days after treatment with DMSO (Bebök et al., 1998). In female mice of both genotypes, DMSO did not significantly alter the response to isoprenaline. Similar observation was made in wild-type male mice. However, in males, DMSO induced a significant increase in isoprenaline-induced salivary secretion in CF male mice. Therefore, we conclude that the method is suitable for testing DMSO-soluble CFTR activators or potentiators administrated by intraperitoneal injection. However, because of a large effect of DMSO in male mice, only female animals were used to evaluate the effects of resveratrol in this study.

In plants, resveratrol exists as the *trans*- and the *cis*- isomers. *Trans*-resveratrol is the primary form found in high abundance in the skin of grapes as well as in many other fruits and vegetables such as berries and peanuts. *Trans*-resveratrol has the largest biological activity and has been extensively investigated. Dozens of investigators have shown that resveratrol can prevent or slowdown the progression of a variety of diseases in animal models and humans. Although its effects on the lifespan of many model organisms remain controversial (Kasiotis et al., 2013), anticancer (Bhat and Pezzuto, 2002), anti-inflammatory (Donnelly et al., 2004) and beneficial cardiovascular (Wu and Hsieh, 2011) effects have been reported. The distribution of polyphenols in organ tissues seems to vary depending on the mode of administration (Planas et al., 2012). In our studies, resveratrol was administrated by intraperitoneal injection; we observed that two hours after administration, very low circulating levels were detected with higher levels being found in the salivary glands. The dose we tested corresponded to the minimal dose that has been shown to be active on CFTR function. We performed the salivary secretion assay two hours after treatment based on previous observation (Hamdaoui et al., 2011) that the effect of resveratrol on F508del-CFTR expression in CF pancreatic cell line CFPAC-1 is stronger after two hours of exposure.

The mechanism of action of resveratrol on CFTR activity is largely unknown and it seems that several pathways could be responsible for the *in vivo* effects. For example, it has been shown that resveratrol displays proteasome- (Qureshi et al., 2012) or cAMP-dependent phosphodiesterases (PDE) (Park et al., 2012) inhibiting effects. In the context of CFTR, proteasome inhibitors have been recently shown to rescue F508del-CFTR function in mouse intestinal tissues (Wilke et al., 2012) and we have demonstrated that inhibitors of PDE5 can act as correctors and potentiators of CFTR mutant proteins (Lubamba et al., 2008). Moreover, resveratrol has also been shown to modulate various epigenetic processes including DNA methylation, histone modification, chromatin remodeling, proteostasis balance and microRNA (miRNA) regulation (reviewed in Wang et al., 2013). Several lines of evidence have indicated that CFTR mutations may alter major signaling pathways through altered expression of miRNAs (Xu et al., 2011) or by modulation of proteostasis network (Balch et al., 2011). As we evaluated effects of resveratrol two hours after injection, it is likely that they are due to acute potentiator effect (as resveratrol induced salivary secretion in wild-type animals as well) and corrector effect rather than epigenetic mechanisms. Our data showed an increased CFTR immunolabelling in human CF and wild-type bronchial epithelial cell lines, suggesting that the increased rate of isoprenaline-induced secretion in salivary glands could be associated with an increased CFTR protein expression. In addition, the redistribution of the fluorescence intensity signal towards cell membranes in both CF and wild-type cells suggests that resveratrol could also act as a corrector. However, longer exposure could alter various epigenetic pathways and be an additional therapeutic benefit for CF.

In conclusion, we showed here that resveratrol partially rescues the refractory CFTR-dependent β-adrenergic response of saliva secretion in female CF mice. Our data indicate that the salivary secretion assay has a potential application to test efficacy of novel CF therapies.

## MATERIAL AND METHODS

### Animal model

C*ftr*^tm1Eur^ mice homozygous for the F508del-CFTR mutation, F508del-CFTR heterozygous and their wild-type homozygous littermates, built in the 129/FVB outbred background (van Doorninck et al., 1995), were housed at the Animal Care Facility of our University following recommendations of the Federation of European Laboratory Animal Science Associations (Nicklas et al., 2002). The experimental protocol was approved by the local Ethics Committee for animal research at the Université Catholique de Louvain (2013/UCL/MD/012) and conformed to the European Community regulations (CEE no. 86/609). To prevent intestinal obstruction in CF mice, Movicol (55.24 g/l; Norgine, Heverlee, Belgium) was administered in acidified drinking water. As human, homozygous F508del mice are infertile; heterozygous mice were then used to maintain the breeding colony. The mice were genotyped at 21 days of age as previously described (Legssyer et al., 2006). Mice aged 2 to 4 months (20–30 g) were anesthetized with a mixture of 10 mg/ml ketamine and 1.25 mg/ml midazolam in sterile NaCl 0.9%. At the end of the experiment, mice returned to their cages after regaining consciousness.

### Administration of resveratrol versus placebo

**Fig. 5.**
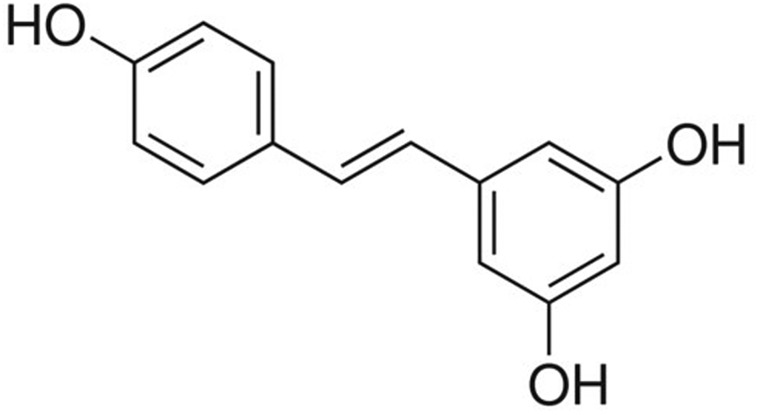
**Structure of *trans*-resveratrol (trans-3,5,4′-trihydroxystilbene, C_14_H_12_O_3_, molecular weight: 228.24 g/mol).** The stilbene core consists of a *trans*-ethane double bond substituted with a phenol group on both carbon atoms of the double bond. It is practically insoluble in non-organic solvents. The three -OH groups of *trans*-resveratrol confer an estimated water solubility of 0.03 g/ml which makes impossible the use of aqueous resveratrol solution in our study.

According to the manufacturer's datasheet, predicted DMSO solubility of resveratrol (Fig. 5) is 16 g/l. The compound was dissolved in DMSO (15 mg/ml) and the solution was diluted 1:1 in NaCl 0.9%. Resveratrol (50 mg/kg) was administered by intraperitoneal injection two hours prior to sampling. In control experiments, vehicle alone (DMSO:NaCl 1:1) was administered (final volume 160 µl).

### Resveratrol quantification assay

Two hours after intraperitoneal injection of resveratrol, wild-type mice were anesthetized with 24 mg pentobarbital. After blood collection by cardiac puncture, the salivary glands were dissected, placed in a preweighed microcentrifuge tube that was then reweighted. Total blood and salivary glands were processed for resveratrol quantification analyses. Organs were homogenized in dH_2_O with an Ultra-Turrax T10 (IKA, Boutersem, Belgium). Resveratrol calibrators were prepared in human plasma, at concentrations ranging from 1 to 250 ng/ml. To 100 μl of sample (plasma, salivary gland tissue homogenates or calibrator), 100 µl of phosphoric acid 4% and 1 ml of methanol containing hexestrol as an internal standard were added. Samples were vortexed for 10 s and centrifuged for 10 min at 10,000 ***g***. The supernatant was evaporated to dryness and reconstituted with 75 µl of 10 mM ammonium formate buffer (solvent A). A volume of 20 µl of each sample and calibrators was injected in a high performance liquid chromatography system coupled with tandem mass spectrometry detection (HPLC-MS/MS, Alliance 2795 from Waters^®^ coupled with a Quattro-micro from Micromass^®^). The mobile phase consisted of methanol and solvent A. Over 0–1.5 min, methanol linearly increased from 20 to 100% and remained at 100% for 4 min, before returning to 20% over 1.5 min. Separation was achieved using a Waters^®^ Xselect HSS C18 column (100Å, 3.5 µm, 2.1×75 mm) and mass spectrometry detection was achieved in negative electrospray ionization mode. The compound mass transitions were 227.08>185.12 (resveratrol), 269.12>134.28 (hexestrol) using multiple monitoring acquisition mode. The HPLC-MS/MS assay was validated according Food and Drug Administration criteria. The assay was linear within the concentration range tested (1–250 ng/ml) with intra- and inter-day precision of <8.6% and <10.02% respectively. The limit of quantification and detection were 1 and 0.5 ng/ml respectively. Calibration curves were built from peak areas ratios to the internal standard (resveratrol) using least-squares linear regression with a (1/concentration^2^) weighting factor that was chosen based on goodness-of-fit criteria. The regression line was then used to calculate sample resveratrol concentrations. For tissue homogenates, concentrations were normalized to express results in ng/mg tissue.

### Salivary secretion assay

The salivary secretion was assayed in F508del-CFTR homozygous, heterozygous and wild-type mice by the method adapted from Best and Quinton (2005). Briefly, anesthetized mice were maintained in supine position. Pharmacological agents (atropine and isoprenaline) were injected subcutaneously in the left cheek and saliva was collected in small pieces (2 mm×25 mm) of Whatman^®^ paper inserted in the mouth near the site of injection. To avoid saturation with saliva, the piece of paper was renewed every 2 min. Each piece was placed in a cap-secure microcentrifuge tube to avoid evaporation. The total collection time was 18 min. At the end of sampling, the net weight of saliva secreted during the collection was determined by measurement in a 0.1 mg resolution analytical scale (Mettler Toledo AE 240, Zaventem, Belgium). In all experiments, basal salivary secretion was measured before any drug injection. The cholinergic-dependent component of salivary secretion was blocked by injection of 50 µl of 1 mM atropine. The remaining atropine-insensitive secretion was stimulated by injection of 50 µl of 100 µM isoprenaline while maintaining cholinergic blockade with atropine (1 mM). Secretory rates were expressed as the weight of saliva per minute of collection. Results were normalized by animal body weight.

### Human bronchial epithelial (HBE) cell lines

Wild-type (16HBE14o−) and CF (CFBE41o−) cell lines [a kind gift of D.C. Gruenert from the University of California, San Francisco, USA; (Illek et al., 2008)] were cultured in Minimal Essential Media (MEM) supplemented with 10% fetal bovine serum, 1% penicillin-streptomycin and 2 mM L-glutamine. HBE cultured cells were incubated for 2 or 24 h with either 100 µM resveratrol dissolved in DMSO or DMSO alone as a negative control.

### RT-qPCR quantification of CFTR transcripts

RNA was extracted with Tripure^®^ Reagent (Roche Diagnostics, Vilvoorde, Belgium), according to manufacturer's instructions. RNA (1 µg) was reverse transcribed using SuperScript^®^ VILO™ cDNA Synthesis Kit (Life Technologies, Merelbeke, Belgium) in a final volume of 20 µl. Resulting cDNA were then diluted in sterile nuclease-free water and used as a template in subsequent real-time PCR analysis. A relative quantification of mRNA expression was performed on an ABI 7000 real-time PCR thermocycler (Applied Biosystems, Gent, Belgium) in the following conditions: 2 min 50°C, 10 min 95°C and 40 cycles of 15 s at 95°C and 1 min at 60°C using TaqMan^®^ Gene Expression Master mix (Life Technologies) in a total volume of 20 µl. Each experimental condition was run in triplicate. Results represents the average of two independent experiments and were expressed as the ratio of the expression of different genes to the reference housekeeping gene 18S RNA. Relative expression (−ΔΔCt) was normalized to the DMSO control.

### Immunohistochemistry in human cell lines

16HBE14o− and CFBE41o− cells grown on glass coverslips were fixed in ice-cold methanol for 10 min at 4°C and blocked for 1 h with 1% BSA in PBS. They were incubated overnight at 4°C with primary anti-CFTR monoclonal antibody raised against the C-terminus (clone 24-1, MAB25031; R&D Systems, Abingdon, UK; dilution 1:100) and primary anti-ZO-1 (zonula occludens-1, PA5-28869; Thermo Scientific, Asse, Belgium; dilution 1:150). After rinsing three times in PBS containing 0.1% Tween 20, cells were incubated for one hour at room temperature with goat anti-mouse [Alexa Fluor 488 IgG (H+L), 2 mg/ml; Invitrogen, Merelbeke, Belgium] and goat anti-rabbit [Alexa Fluor 555 IgG (H+L), 2 mg/ml; Invitrogen] secondary antibody, both diluted 1:1000 in PBS containing 0.1% Tween 20. Cells were washed three times before being mounted on slides with anti-fading medium containing DAPI (SlowFade Gold antifade reagent with DAPI, Life Technologies). Slides were then stored at 4°C in the dark and imaged by microscopy using a Zeiss AxioImager Z1 fluorescent microscope. Images were taken with exposition time of 200 ms using a 20× magnification. Images were exported to AxioVision Release 4.9.1 software. Cross-sectional scans of pixel intensities of individual cells were measured at their largest diameter and analyzed using AxioVision software (version 4.9.1). The mean CFTR-related intensity across each cell (*n*=8 cells per condition, randomly selected) was measured as representative of CFTR distribution.

### Chemicals

Atropine, isoprenaline hydrochloride and *trans*-resveratrol (Sigma Aldrich, Diegem, Belgium) were diluted in a filtered 0.9% NaCl solution except resveratrol which was dissolved in a mixture of DMSO: 0.9% NaCl (1:1).

### Data analysis and statistics

Data obtained from the salivary secretion tests and from HPLC-MS-MS analytical assays are presented as median (25–75% interquartile range), where *n* refers to the number of animals per group. Based on smaller sample sizes, between-group comparisons were evaluated by nonparametric analysis using median rank scores. When not specified otherwise, data are expressed as mean±s.e.m. after checking normality of distribution including the Shapiro-Wilk test. Between-group comparisons of normally distributed data were evaluated by using one-way analysis of variance with posthoc comparisons being made using Student *t*-test or Tukey-Kramer honestly significant difference test for two or more than two x-levels, respectively. Null hypothesis was rejected at *P*<0.05. Graphs and scatterplots were drawn using GraphPad prism 6.0 for Windows (GraphPad Software, San Diego, CA, USA).

## Supplementary Material

Supplementary Material
