# Supplementary material for: Resveratrol increases F508del-CFTR dependent salivary secretion in cystic fibrosis mice
Source: Biol Open. 2015 Jun 19;4(7):929–36. doi: 10.1242/bio.010967 (PMC4571083; doi:10.1242/bio.010967)
Supplement: Supplementary Material [file supp_4_7_929__index.html]

Resveratrol increases F508del-CFTR dependent salivary secretion in cystic fibrosis mice — Resveratrol increases F508del-CFTR dependent salivary secretion in cystic fibrosis mice — Supplementary Material 

# Resveratrol increases F508del-CFTR dependent salivary secretion in cystic fibrosis mice

## BIO010967 Supplementary Material

- Supplementary Material
