# Supplementary material for: Resveratrol increases F508del-CFTR dependent salivary secretion in cystic fibrosis mice
Source: Biol Open. 2015 Jun 19;4(7):929–36. doi: 10.1242/bio.010967 (PMC4571083; doi:10.1242/bio.010967)
Supplement: Supplementary Material [file supp_bio.010967_BIO010967supp.pdf]

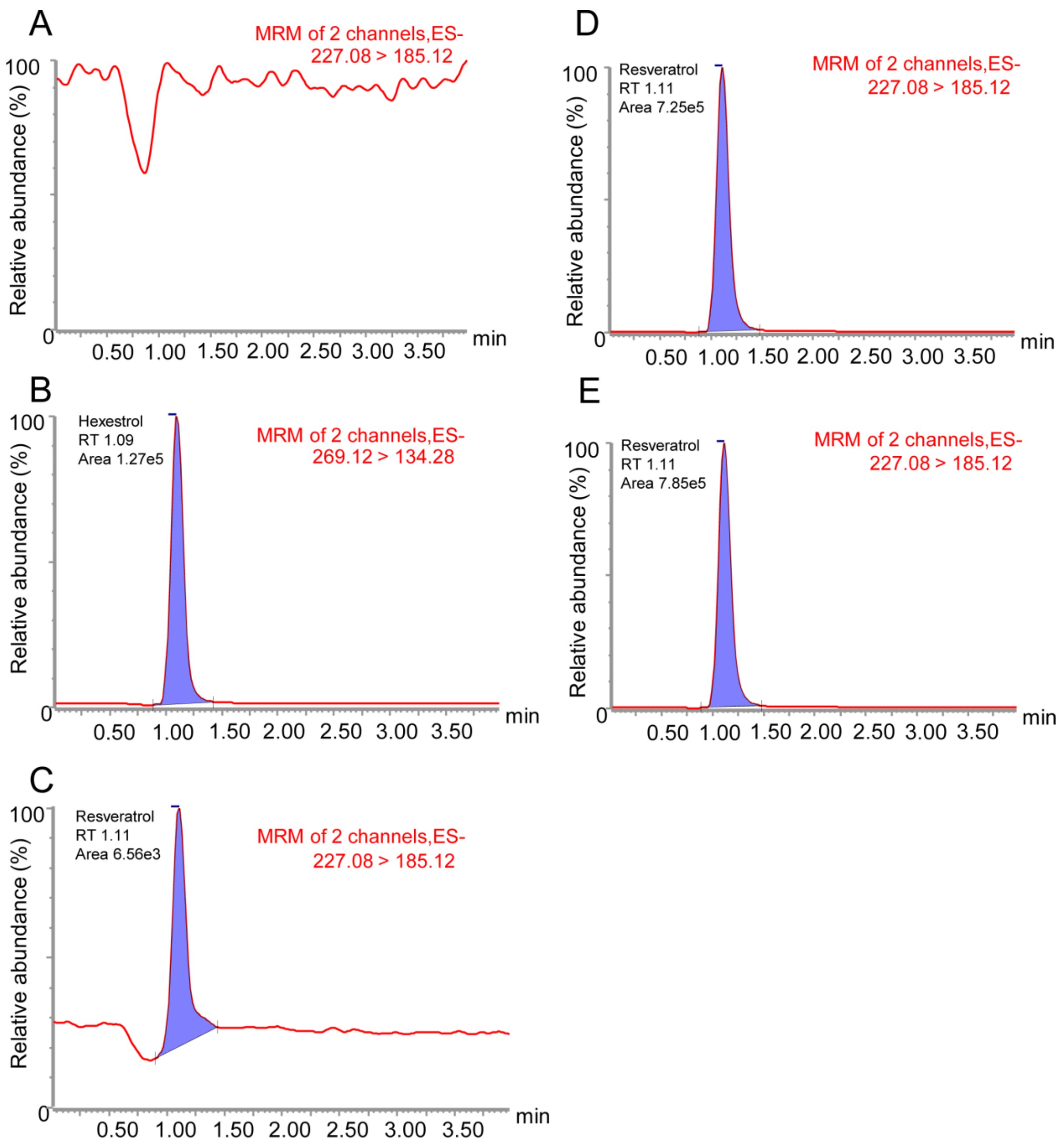

Fig. S1: Typical ion chromatograms of sample extracts obtained from a blank sample (A), an internal standard (IS) (B) or from a sample spiked at the 1ng/ml. Limit of quantification (LOQ) of resveratrol (C). (D) and (E) represent blood and extracts of a sample of salivary glands at 26.6 ng/mL and 27.4 ng/mg resveratrol respectively (RT= Retention time). Y axis given in terms of relative abundance. The base peak is the ion with the highest measured abundance. Its relative abundance is assigned a value of 100, and the abundances of all the other plotted ions are normalized to that value. MRM of 2 channels, ES-: Multiple Reaction Monitoring in 2 MS/MS channels of the two different precursor–product ion pairs, resveratrol and hexestrol, in Negative Electrospray ionization.
